# Cancer-Testis Antigen LDH-C4 in Tissue, Serum, and Serum-Derived Exosomes Serves as a Promising Biomarker in Lung Adenocarcinoma

**DOI:** 10.3389/fonc.2022.912624

**Published:** 2022-06-24

**Authors:** Wei Peng, Jin Chen, Yanping Xiao, Guangjian Su, Yan Chen, Zhaolei Cui

**Affiliations:** Laboratory of Biochemistry and Molecular Biology Research, Fujian Key Laboratory of Advanced Technology for Cancer Screening and Early Diagnosis, Department of Clinical Laboratory, Fujian Medical University Cancer Hospital, Fujian Cancer Hospital, Fuzhou, China

**Keywords:** cancer-testis antigen, lactate dehydrogenase C4, lung adenocarcinoma, exosome, diagnosis, prognosis

## Abstract

**Objective:**

As a cancer-testis antigen (CTA), human lactate dehydrogenase C4 (LDH-C4) enzyme protein encoded by the *LDHC* gene has been reported to be involved in the occurrence and development of various malignancies, while its expression and clinical significance in lung adenocarcinoma (LUAD) remain unclear. This study aims to investigate the expression of LDH-C4 in LUAD and its diagnostic and prognostic value.

**Methods:**

The mRNA and protein levels of LDH-C4 in LUAD and adjacent normal tissues were analyzed based on the UALCAN database, and the prognostic significance was assessed using the LOGpc database. The *LDHC* mRNA level in serum and serum secretion of LUAD patients was determined by quantitative real-time PCR (qRT-PCR). Based on the high-throughput LUAD tissue chip combined with immunohistochemistry (IHC), the protein level of LDH-C4 in LUAD tissues was measured, and its correlation with clinicopathological features and prognosis was analyzed.

**Results:**

*LDHC* expression was upregulated in LUAD, which was related to the clinical stage and poor prognosis of patients. The positive rates of *LDHC* mRNA expression in serum and exosome of LUAD patients were 78.3% and 66.7%, respectively. The area under the curve (AUC) of serum and exosomal *LDHC* in the diagnosis of LUAD was 0.8121 and 0.8925, respectively. The expression of *LDHC* in serum and serum-derived exosomes from LUAD patients was negatively correlated with medical treatment and positively correlated with the recurrence of LUAD. The positive expression rate of LDH-C4 in LUAD tissues was 96.7% (89/92), which was significantly higher than that in adjacent normal tissues 22.6% (19/84) (*p* < 0.001). The median overall survival (OS) time of patients with a high expression of LDH-C4 was significantly shorter than that of patients with low expression (34 months versus 62 months) (*p* = 0.016). Further relative risk analysis exhibited that the expression of LDH-C4 was an independent prognostic factor of OS in patients with LUAD.

**Conclusions:**

*LDHC*/LDH-C4 expression was upregulated in LUAD, and LDH-C4 could be used as a molecular indicator of the prognosis of LUAD. Serum and serum-derived exosomes of *LDHC* can be used as an important biomarker for the diagnosis, efficacy evaluation, and recurrence monitoring of LUAD.

## Introduction

Lung cancer is the leading cause of cancer-related mortality worldwide (1). Non-small cell lung cancer (NSCLC) accounts for approximately 85% of all lung cancer types, of which lung adenocarcinoma (LUAD) is a striking pathological type (2). It has been reported that LUAD accounts for about 38.5% of the incidence rate of lung cancer (3). This type has a shorter metastasis time, a shorter course of disease, and faster invasion and metastasis than lung squamous cell carcinoma, which are the main causes of death in LUAD patients (4). With the advancement of molecular targeted therapy and immunotherapy (5, 6), the prognosis of LUAD has been improved, while the prognosis remains poor as a large number of LUAD still lack specific gene targets without available specific targeted drugs (7). Therefore, there is an urgent need to explore novel therapeutical targets for LUAD, which is of great significance for further molecular targeted therapy.

Cancer-testis antigens (CTAs), a family of tumor-associated antigens, are a kind of antigen only expressed in testicular germinal epithelium and some tumor tissues, which is one of the potential markers of tumor diagnosis and immunotherapy (8, 9). Lactate dehydrogenase C4 (LDH-C4) protein (also named LDHC or LDH-X), known as an important CTA (10), is the sixth LDH isoenzyme only expressed in mammalian testis and spermatozoa, but not expressed in normal somatic tissues and cells (10, 11). Numerous studies have attempted to explain the roles of *LDHC*/LDH-C4 in the occurrence and development of multiple human cancers, such as renal cell carcinoma (12), hepatocellular carcinoma (HCC) (13), and breast cancer (BC) (14). In our previous work, it is interesting to note that the positive expression of LDH-C4 is significantly correlated with the decreased survival rate and the increased risk of poor clinical prognosis of the cancer cases (13, 14). Immunohistochemical staining showed that LDH-C4 is positively expressed in BC cell MDA-MB-231, which is mainly localized in the cytoplasm (15). We also found that the positive expression rates of *LDHC* mRNA in serum and serum-derived exosomes of BC patients were 91.66% and 87.50%, respectively, and that circulating *LDHC* can be used for the diagnosis, efficacy evaluation, and recurrence monitoring of BC (14). In HCC, the positive rates of *LDHC* expression in serum and serum-derived exosomes are 68% and 60%, respectively; the expression of *LDHC* in serum and serum-derived exosomes in patients receiving treatment was significantly lower than that in the initial diagnosis group; the high expression of LDH-C4 is correlated with the poor prognosis of patients, suggesting an independent risk factor for the prognosis of patients with HCC (13). Recent evidence demonstrated that excessive activation of *LDHC* in A549 LUAD cells increased the phosphorylation levels of AKT and GSK-3β, revealing the activation of the PI3K/Akt/GSK-3β oncogenic-signaling pathways (16). However, the expression and clinical significance of *LDHC*/LDH-C4 in lung cancer remain to be elucidated.

A prior study has revealed that among the seven detected CTAs, *LDHC* is the only molecule with a positive expression in all types of pathological tissues of non-small-cell lung cancer (NSCLC), suggesting that *LDHC* acts as a potential marker for the diagnosis of NSCLC (11). This prompted us to hypothesize that *LDHC*/LDH-C4 may be utilized as novel diagnostic and prognostic biomarkers in LUAD. The current study was conducted with the central objective of the expression and clinical significance of tissue *LDHC*/LDH-C4 in LUAD based on bioinformatics analysis of the online databases. We further investigated the expression of *LDHC*/LDH-C4 in different test matrices (tissue, serum, and serum-derived exosomes) of the LUAD cases using qRT-PCR, IHC, and immunoblotting.

## Materials and Methods

### Study Design

**Figure 1:**
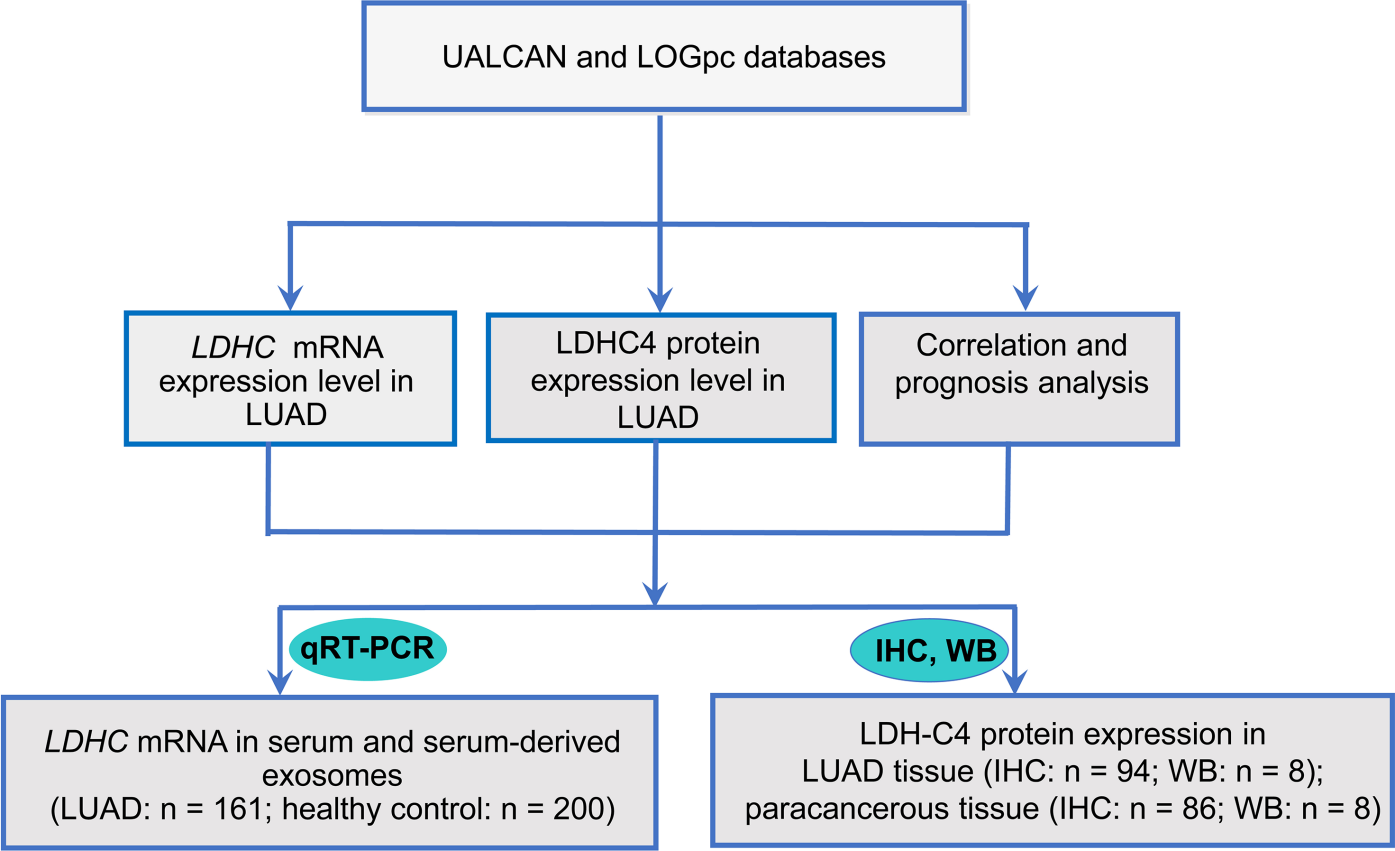
Flowchart of research design. IHC, immunohistochemistry; qRT-PCR, quantitative real-time PCR; WB, Western blotting.

As shown in **Figure 1**, the UALCAN database was adopted to analyze the mRNA and protein levels of LDH-C4 in LUAD and adjacent normal tissues. The correlation between *LDHC* expression and prognosis of LUAD was analyzed by the LOGpc database as well. Based on bioinformatics analysis, IHC and immunoblotting were performed to verify the expression of LDH-C4 in LUAD and its correlation with clinicopathological features and prognosis. The *LDHC* mRNA level in serum and serum-derived exosomes from LUAD patients was detected by qRT-PCR to further verify the reliability of bioinformatics analysis.

### Public Data and Tools

The *LDHC* expression in LUAD and adjacent normal tissues was determined by the UALCAN (17). The prognosis of patient LUAD was analyzed by the LOGpc platform with comprehensive data sources and a large sample size, including 2,277 malignant tumors, 31,310 patients, and 209 datasets from multiple databases such as TCGA, GEO, and CGGA (18). The correlation between *LDHC* expression and the prognosis of LUAD was also analyzed based on LOGpc, and the forest maps of hazard ratios (HRs) from different datasets were combined.

### Clinical Data

Based on the established clinical data of the HLugA180Su05 high-throughput tissue chip (SHANGHAI OUTDO BIOTECH, China), 94 LUAD patients (92 LUAD patients and 2 patients with lost follow-up information) and 86-paired paracancerous tissues from July 2004 to April 2009 were enrolled in this study. The clinical data of the high-throughput tissue chip included patient age, gender, tumor size, clinical stage, and EGFR mutation, among others. The follow-up time was up to August 2014. The tissue chip has obtained the corresponding ethical approval of SHANGHAI OUTDO BIOTECH. In addition, serum from 161 LUAD patients and 200 healthy individuals in Fujian Cancer Hospital from March 3, 2020 to November 30, 2021 was collected. All serum samples were collected with the approval of the ethics committee of Fujian Cancer Hospital (No. SQ2019-012-01). The clinical staging of all included LUAD cases was confirmed in line with the American Joint Committee on Cancer (AJCC) seventh edition cancer staging manual.

### IHC Assay

One high-throughput tissue chip (HLugA180Su05, each tissue chip contains 94 points of LUAD tissues and 86 points of corresponding adjacent normal tissues) was selected. For this experiment, the EliVisionTM Plus (MXB Biotechnologies, Fuzhou, China) two-step detection system was used, and the rabbit monoclonal anti-human LDHC primary antibody (Abcam, cat. No. ab52747; 1:100 dilution) served as the primary antibody according to the instructions of the En Vision DAB detection kit (MXB Biotechnologies, Fuzhou, China). The 4-μm paraffin-embedded tissues fixed with formaldehyde were analyzed by IHC. PBS instead of primary antibody functioned as the negative control and observed under the optical microscope (Olympus BX40; Tokyo, Japan). The sections were scored according to the degree of color (13): sections without immune color development were regarded as 0 point, sections with light brown as 1 point, sections with medium brown as 2, and sections with dark brown as 3. According to the proportion of positive staining of sections, the sections were scored as follows: 0 for <5%, 1 for 5%–25%, 2 for 25%–50%, and 3 for >50%. The final score of LDH-C4 expression is the product of score and intensity: 0 is -, 1–2 is +, 3–5 is ++, and 6–9 is +++, in which “-”/”+” refers to low expression and “++”/”+++” refers to high expression.

### Isolation and Identification of Exosomes

ExoRneasy Serum/Plasma Midi Kit (QIAGEN, Catalog No.77044) (Part I: vesicle isolation) was employed to extract exosomes from serum, which was conducted according to www.qiagen.com/HB-1179. In detail, the specimen was centrifuged at 4°C and 16,000 × *g* for 10 min, and the particles larger than 0.8 μm in diameter were filtered out; 500 μl of serum was mixed with buffer XBP at a ratio of 1:1. The serum/buffer XBP mixture was added to an exoEasy spin column, and centrifuged at 500 × *g* for 1 min. Subsequently, 3.5 ml of buffer was added, and centrifuged at 5,000 × *g* for 5 min. Afterwards, 700 μl of QIAzol was added to the film, and centrifuged at 5,000 × *g* for 5 min to collect dissolved solution and completely transferred to a 2-ml matched test tube. The extracted exosomes were identified by a transmission electron microscope (TEM) (Hitachi TEM system). Total protein was extracted from the obtained exosomes by RIPA lysate. The concentration of antibody is 1:100 for monoclonal antibody mouse anti-human GAPDH and 1:100 for monoclonal antibody rabbit anti-human CD63 (Abcam, catalog No.ab217345) and CD9 (Abcam, catalog No.ab92726) primary antibodies. Cytochrome C (Cyto C) (mitochondrial marker; 1:50 dilution; Abcam, rabbit monoclonal [EPR1327] to Cyto C; catalog No. ab133504) served as a negative control to exclude the possible mixture of cellular contamination (19).

### Quantitative Real-Time PCR

The total RNA was isolated from serum using the MiRNeasy Kit (QIAGEN, catalog No. 217184) as our published paper described (19). In detail, 200 μl of serum sample was taken and 1 ml of QlAzol Lysis buffer was added, then stirred at room temperature for 5 min; 200 μl of chloroform was added, shaking for 15 s and incubating at room temperature for 2 to 3 min; the mixture was centrifuged at 4°C and 12,000 rpm for 15 min; the supernatant was transferred to a new EP tube, and 1.5 times volume of 100% ethanol was added. After sucking and mixing evenly, 700 μl of mixture was transferred to a new 2-ml tube with RNeasy MinElute nucleic acid purification column, centrifuged at 8000 × *g* and at room temperature for 15 s; 700 μl of buffer RWT was added, centrifuged at 8,000 × *g* and at room temperature for 15 s, and the waste liquid was discarded; then, 500 μl of buffer RPE was added, centrifuged for 15 s under the same conditions, and the waste liquid was discarded; 500 μl of 80% ethanol was added, centrifuged for 2 min, the liquid and tube were discarded, and the nucleic acid separation column was left behind; the RNeasy MinElute nucleic acid purification column was added into a 2-ml new tube, centrifuged at full speed until dry; the nucleic acid separation column was transferred to a new 1.5-ml tube, and 14 μl of RNase distilled water was added to the center of the nucleic acid purification column film; and the cover was closed, centrifuged at full speed for 1 min.

For the RNA isolation of exosomes [following the above ExoRneasy Serum/Plasma Midi Kit (Part I: vesicle isolation)], 90 μl of chloroform was added, and the tube cover was closed, shaken forcibly for 15 s, and incubated at room temperature for 2 to 3 min; the upper water phase was transferred to a new collection tube by centrifugation at 4°C and 12,000 × *g* for 15 min. Double volume of 100% ethanol was added and mixed evenly. The sample larger than 700 μl was pipetted into an RNeasy MinElute spin column in a 2-ml collection tube, centrifuged at room temperature and ≥8,000 × *g* for 15 s, and the waste liquid was discarded. Afterwards, 700 μl of RWT buffer was added to the RNeasy MinElute spin column, centrifuged at room temperature and ≥8,000 × *g* for 15 s, and the waste liquid was discarded. Next, 500 μl of buffer RPE was added to RNeasy MinElute spin column, then centrifuged at room temperature ≥8,000 × *g* for 15 s, and the waste liquid was discarded. Then, 500 μl of buffer RPE was added to the RNeasy MinElute spin column, and the tube cover was covered and centrifuged at room temperature ≥8,000 × *g* for 2 min. Subsequently, the RNeasy MinElute spin column was placed in a new collection tube, and the lid of the spin column was opened and centrifuged at full speed for 5 min to dry the film; the RNeasy MinElute spin column was placed in a new matched 1.5-ml collection tube, and 14 μl of RNase-free water was directly added to the center of the spin column film. The column stood for 1 min, and then centrifuged at full speed for 1 min to extract RNA and determine the purity of total RNA.

The extracted RNA was reversely transcribed into cDNA by the Transcriptor First strand cDNA synthesis kit (Roche). Subsequently, the target product was amplified by SYBR Green Master (ROX). The primer sequences for *LDHC* and *GAPDH* (internal reference gene) were documented in our published studies (13, 14): *LDHC*-F: 5′-TCATTCCTGCCATAGTCCA-3′, *LDHC*-R: 5′-CAATTACACGAGTTACAGGTA-3′, *GAPDH*-F: 5′-TCGACAGTCAGCCGCATCTTCTTT-3′, and *GAPDH*-R: 5′-ACCAAATCCGTTGACTCCGACCTT-3′. The reaction system was placed in ABI7500 fluorescence quantitative PCR detector for amplification. The conditions were as follows: denaturation at 95°C for 10 min, 95°C for 15 s for 40 cycles, and finally at 60°C for 1 min. The 2^−ΔΔCt^ method was used to quantify the relative expression of target genes.

### Statistical Methods

Data analysis was performed using SPSS 16.0 software (SPSS, Inc., Chicago, IL, USA). All quantitative data are presented as mean ± standard deviation. Comparison of data conforming to normal distribution and homogeneity of variance between the two groups was analyzed by Student’s *t*-test. Comparison of the positive expression rate of LDH-C4 in LUAD tissues and adjacent normal tissues, and the relationship between LDH-C4 expression in tumor tissues and the clinicopathological characteristics of LUAD patients were analyzed using the *χ*^2^ inspection Kaplan–Meier method or Spearman’s correlation analysis, followed by survival analysis using Log rank test. The relative risk analysis was utilized to analyze the effect of LDH-C4 expression and other pathological features on LUAD. Inspection level is set as *α* = 0.05 and values of *p* < 0.05 were considered significant.

## Results

### *LDHC/*LDH-C4 Expression in Lung Cancer Based on Online Database

**Figure 2:**
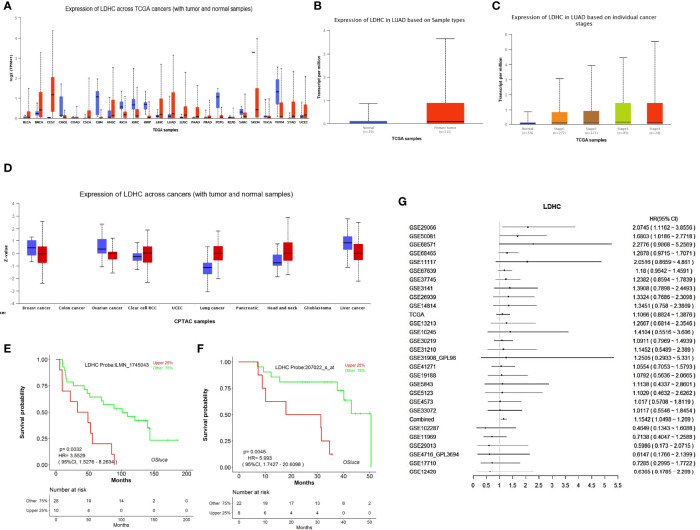
The expression and prognostic value of *LDHC*/LDH-C4 in LUAD were analyzed based on an online database. **(A)** Pan-cancer view of *LDHC* mRNA in the UALCAN database. Expression of *LDHC* in LUAD tissue based on **(B)** sample types (LUAD vs. normal control) and **(C)** clinical stages in UALCAN. **(D)** Pan-cancer review of LDH-C4 protein expression using UALCAN, and all lung cancer samples were from LUAD cases (*n* = 111). The plotted survival curves for **(E)** OS and **(F)** PFS were drawn by the LOGpc database. **(G)** HR forest map of *LDHC* expression and lung cancer prognosis analyzed using the LOGpc platform (combined HR = 1.1542).

Pan-cancer analysis showed that the *LDHC* mRNA level in pan-cancer was higher than that in normal controls, including lung cancer (LUAD and LUSC) (**Figure 2A**). UALCAN analysis exhibited that *LDHC* mRNA in LUAD tissues was higher than that in adjacent normal tissues (**Figure 2B**), which was related to the clinical stage of patients, and the lower expression of *LDHC* indicated the earlier stage of LUAD (**Figure 2C**). In the protein level, LDH-C4 was elevated in pan-cancer versus normal controls, including lung cancer (also cases were LUAD) (**Figure 2D**).

Based on LOGpc prognostic analysis, patients with upregulated *LDHC* displayed poor prognosis, including overall survival (OS) (**Figure 2E**) and progression-free survival (PFS) (**Figure 2F**), suggesting that *LDHC*/LDH-C4 served as an important biomarker for the prognosis of patients with LUAD. The combined HR of *LDHC* in predicting the survival of lung cancer was estimated to be 1.1542 (95% CI: 1.0498–1.2690) (**Figure 2G**).

### *LDHC* Expression in Serum and Serum-Derived Exosomes of LUAD Patients and Its Correlation With Clinicopathological Features

**Figure 3:**
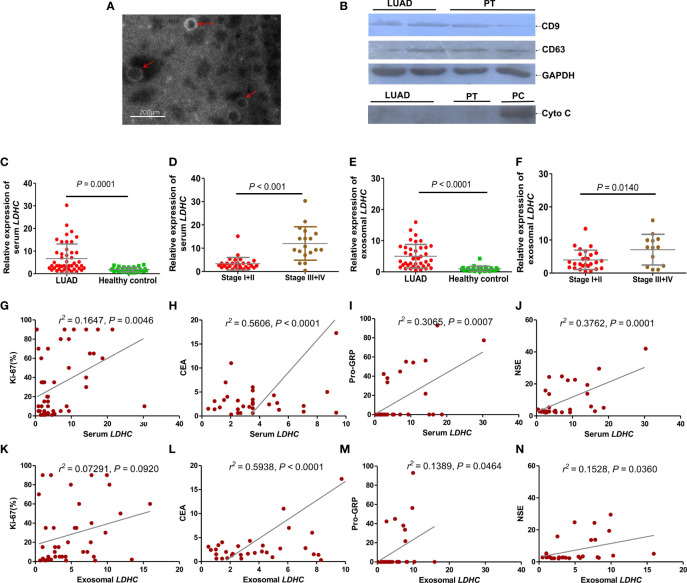
Expression of LDHC in serum and serum-derived exosomes of patients with LUAD and its correlation with clinical molecular markers. **(A)** Identification of extracted serum-derived exosome by transmission electron microscopy. The isolated vesicles were indicated by the red arrows. **(B)** Immunoblotting was used to detect the expression of exosome-related marker proteins. Cyto C (mitochondrial marker) was taken as a negative control to exclude the possible mixture of cellular contamination. PT: paracancerous tissue; PC: positive control using the extracted protein of *LDHC* downregulated A549 cells. The expression of **(C, D)** serum and **(E, F)** serum-derived exosomal *LDHC* in newly diagnosed LUAD patients and patients with different clinical stages were detected by qPCR. Serum *LDHC* was positively correlated with the expression of **(G)** Ki67, **(H)** CEA, **(I)** Pro-GRP, and **(J)** NSE in LUAD patients. Serum-derived exosomal *LDHC* expression was not correlated with **(K)** Ki67, but positively correlated with **(L)** CEA, **(M)** Pro-GRP, and **(N)** NSE.

The morphology and size of the exosomes were identified by TEM. **Figure 3A** shows that the exosome derived from serum is a small vesicle with a membrane structure, with a size of 30 to 150 nm, which is in line with the overall morphological characteristics of exosomes. The expression of exosome marker proteins CD9 and CD63 was assessed by immunoblotting, which exhibited that the expression of CD9 and CD63 could be detected in the exosomes, indicating that serum-derived exosomes from LUAD patients were successfully isolated (**Figure 3B**). The pollution of cell components in exosomes was further eliminated by determining the expression of cytochrome C (Cyto C) (**Figure 3B**). qRT-PCR showed that the positive rates of *LDHC* expression in serum and serum-derived exosomes in LUAD patients were 78.3% (47/60) and 66.7% (40/60), respectively, while the positive rates of *LDHC* expression in serum and serum-derived exosomes in healthy individuals were 15% (30/200) and 14% (28/200), respectively. The expression of *LDHC* in serum and serum-derived exosomes was markedly elevated in LUAD cases than healthy individuals (**Figures 3C, E**). Moreover, *LDHC* levels in serum and serum-derived exosomes were increased with the progression of LUAD, in which the cases in late clinical stages yielded more higher *LDHC* levels than these patients with early stage (**Figures 3D, F**). Correlation analysis showed that *LDHC* expression in serum was positively correlated with the expressions of Ki67, CEA, Pro-GRP, and NSE (**Figures 3G–J**). Similarly, *LDHC* expression in serum-derived exosomes was also found to be positively correlated with the expressions of CEA, Pro-GRP, and NSE except Ki67 (**Figures 3K–N**). Data characteristics of the included LUAD patients are shown in **Table 1**.

**Table 1:** Clinical characteristics of the included LUAD cases for the analysis of serum and exosomal *LDHC* mRNA expressions.

Clinicopathological features	Newly diagnosed LUAD(*n* = 60)		Clinically treated LUAD(*n* = 60)		Recurrent LUAD(*n* = 41)
Age (years)					
>60	30		34		20
≤60	30		26		21
Gender					
Male	39		50		31
Female	21		10		10
T stage					
T1–T2	40		42		12
T3–T4	20		18		29
Clinical stage					
I–II	31		24		2
III–IV	29		36		39
EGFR mutation	7		2		2

### Value of Serum and Exosomal *LDHC* in Diagnosis, Efficacy Evaluation, and Recurrence Monitoring of LUAD Patients

**Figure 4:**
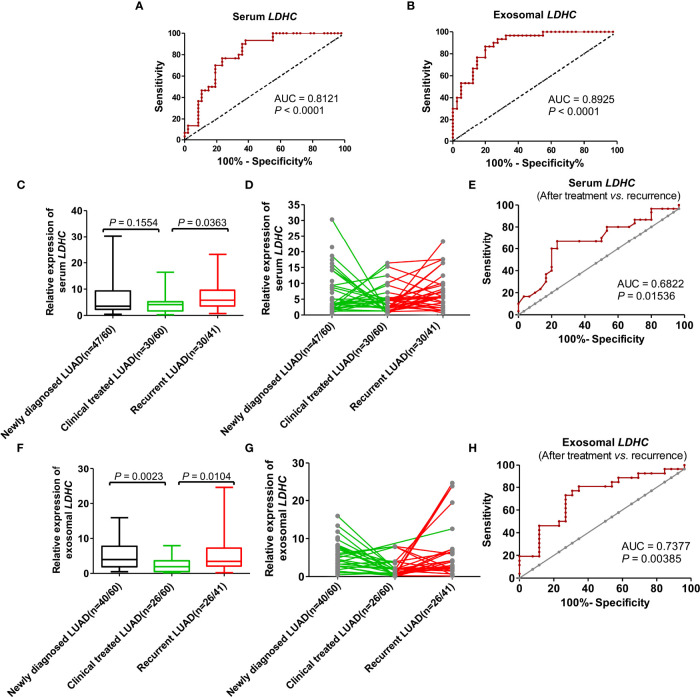
The value of serum and serum-derived exosomal *LDHC* in diagnosis, efficacy evaluation, and recurrence monitoring of LUAD. ROC curve showed the diagnostic efficacy of **(A)** serum and **(B)** exosomal *LDHC* in distinguishing LUAD patients from healthy controls. The expression of **(C, D)** serum *LDHC* in patients with LUAD at first visit, after treatment and relapse. **(E)** The efficacy of serum *LDHC* in predicting recurrence in patients with LUAD after treatment. Expression of **(F, G)** exosomal *LDHC* in patients with recurrent LUAD. **(H)** The efficacy of exosomal *LDHC* in predicting recurrence in patients with LUAD after treatment.

ROC curve analysis showed that serum *LDHC* had a sensitivity of 77%, a specificity of 76%, and an area under the curve (AUC) of 0.8121 (*p* < 0.0001, Youden index = 0.53) (**Figure 4A**). The sensitivity, specificity, and AUC of exosomal *LDHC* were estimated to be of 87%, 80%, and 0.8925 (*p* < 0.0001, Youden index = 0.67), respectively (**Figure 4B**). These findings revealed that serum and exosomal *LDHC* can be used as promising indexes in the diagnosis of LUAD. We also detected the changes of *LDHC* expression in serum and serum-derived exosomes from LUAD patients before and after treatment, which displayed that the serum level of *LDHC* in patients receiving treatment was not significantly altered when compared with the newly diagnosed patients (*p* = 0.1554) (**Figure 4C**). Nevertheless, the average level of *LDHC* in the serum from patients suffering relapses was significantly higher than that in patients receiving treatment (*p* = 0.0363) (**Figures 4C, D**), and the AUC for predicting recurrence was 0.6822 (**Figure 4E**). Moreover, the level of *LDHC* in serum-derived exosome in the newly diagnosed patients was significantly higher than that in patients receiving treatment (*p* = 0.0023) (**Figure 4F**), and the *LDHC* expression in the serum-derived exosome from patients suffering from relapses was significantly higher than that in patients receiving treatment (*p* = 0.0104) (AUC for predicting recurrence = 0.7377, **Figures 4F–H**), suggesting that the expression of serum and exosomal *LDHC* can be used as a good index for predicting the therapeutic effect and recurrence of LUAD.

### LDH-C4 Expression in LUAD Tissues and Its Prognostic Significance

**Figure 5:**
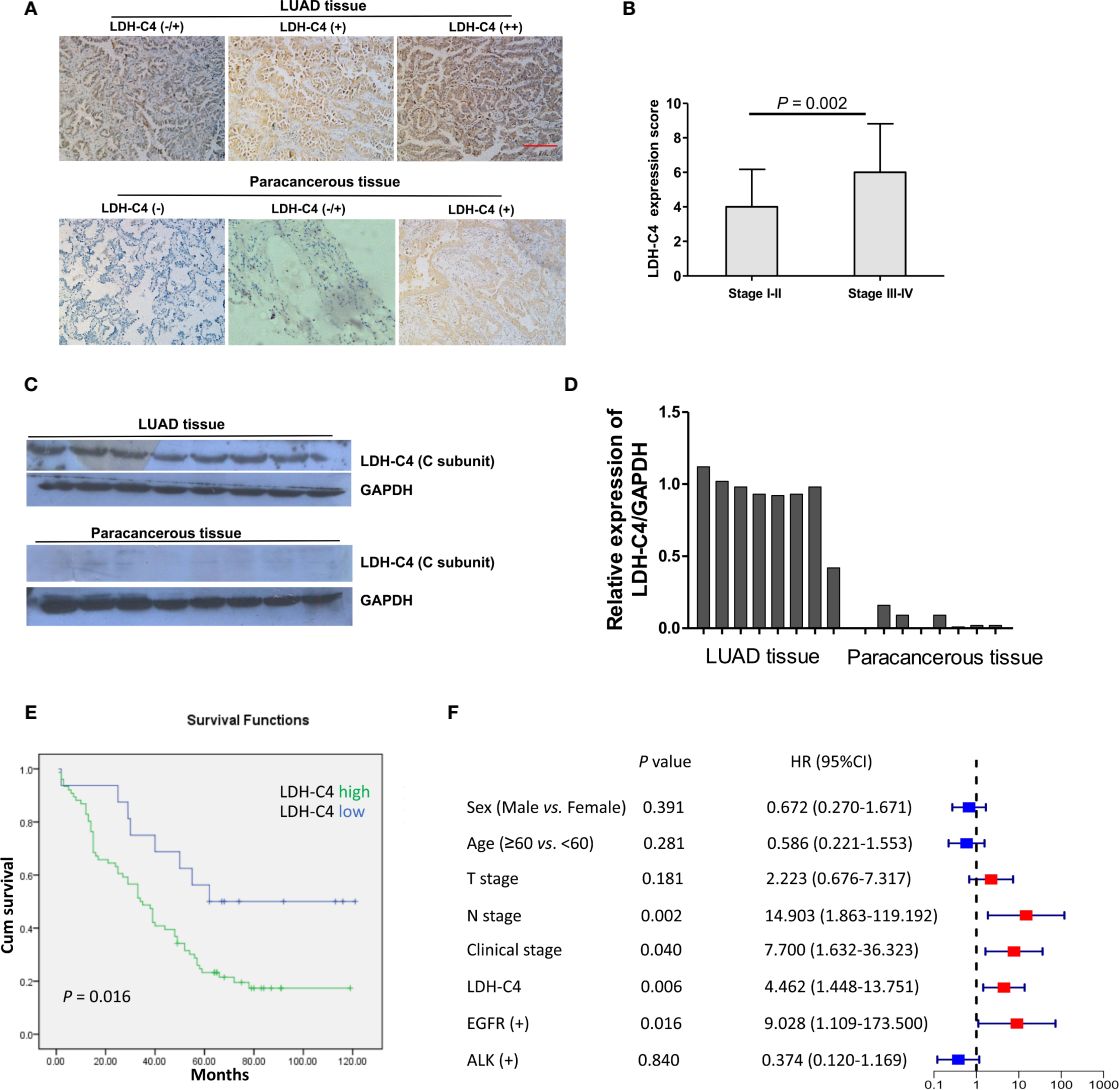
Expression of LDH-C4 protein in LUAD and its prognostic value. **(A)** The expression of LDH-C4 in LUAD and adjacent tissues was detected by IHC analysis based on tissue microarray. **(B)** The expression score of LDH-C4 protein in LUAD patients with stage I + II was lower than that in LUAD patients with stage III + IV. **(C, D)** Immunoblotting was used to detect the expression of LDH-C4 in LUAD patients and matched paracancerous tissues (*n* = 8). **(E)** Patients with high expression of LDH-C4 showed poorer overall survival time. **(F)** The 5-year relative risk analysis based on clinicopathological parameters of LUAD patients.

The IHC analysis presented that LDH-C4 protein was mainly expressed in the cytoplasm of LUAD cells (**Figure 5A**). The detected 92 LUAD cases included 76 cases with LDH-C4 high expression and 16 cases with low expression (3 of them were LDH-C4 negative). The positive expression rate of LDH-C4 in LUAD was 96.7% (89/92), whereas the positive rate in adjacent normal tissues was 22.6% (19/84). The positive expression rate in LUAD tissues was significantly higher than that in adjacent normal tissues (*p* < 0.001). Moreover, LDH-C4 expression (rating scores) was lower in patients with stage I–II than those with stage III–IV (*p* = 0.002; **Figure 5B**). However, it was also found that LDH-C4 expression in LUAD tissues had no correlation with patients’ age, gender, tumor size, lymph node metastasis, clinical stage, and EGFR gene mutation (all *p* > 0.05, **Table 2**). Immunoblotting analysis further confirmed the higher expression levels of LDH-C4 in LUAD tissues, but lower or even absent expression in paracancerous tissues (**Figures 5C, D**). As of the last follow-up date, the median follow-up time of 92 patients with LUAD was 39 months (1 to 121 months). The median OS time of patients with a high expression of LDH-C4 and with low expression was 34 months and 62 months, respectively (*χ*^2^ = 5.810, *p* = 0.016; **Figure 5E**). The 5-year survival rates of patients with a high expression of LDH-C4 and with low expression were estimated to be 22.37% and 56.25%, respectively. The 5-year relative risk analysis exhibited that the high expression of LDH-C4 was 4.462 times the risk of death in patients with low expression (**Figure 5F**), suggesting that LDH-C4 functioned as an independent factor affecting OS in patients with LUAD (all *p* < 0.05).

**Table 2:** Correlations between LDH-C4 protein expressions and clinical characteristics in LUAD patients.

Clinicopathological features	Total case size(*n* = 92)	LDH-C4 low(*n* = 16)		LDH-C4 high(*n* = 76)		*χ*^2^ value	*p-*value
Gender						0.704	0.402
Male	49	7		42			
Female	43	9		34			
Age (years)						2.216	0.137
>60	42	10		32			
≤60	50	6		44			
T stage						0.000	1.000
T1–T2	69	12		57			
T3–T4	23	4		19			
Lymphatic metastasis						0.654	0.419
N0–N1	53	10		43			
N1–N2	22	6		16			
Nx	17						
Clinical stage						0.222	0.638
Stage I+II	43	10		33			
Stage III+IV	32	6		26			
EGFR mutation						0.103	0.749
Negative	73	13		60			
Positive	14	2		11			
Unclear information	5						
ALK mutation						0.085	0.771
Negative	77	13		64			
Positive	15	3		12			

The final score of LDH-C4 expression is the product of score and intensity.

## Discussion

LUAD, as the most universal type of lung cancer, is characterized by rapid metastasis and a short course of disease (1, 3, 4). Invasion and metastasis are the main reasons for mortality in LUAD patients (20). Advancements in pathophysiological understanding have increased the array of treatment options for local and advanced disease, leading to individual treatment plans. Advancements in pathophysiological understanding of molecular targeted therapy and immunotherapy (5), such as epidermal growth factor receptor (EGFR) and anaplastic lymphoma kinase (ALK), have improved the prognosis of LUAD patients (21), while a considerable number of LUAD still lack specific targets. Previous studies have shown that LDH-C4, as an important tumor CTA, plays an important role in tumorigenesis and development (10–15, 19), acting as a potential target for tumor immunotherapy (22). In this research, we thoroughly analyzed the expression of *LDHC*/LDH-C4 in different matrix of LUAD as well as the efficacy of *LDHC*/LDH-C4 expression as a biomarker in diagnosis, efficacy evaluation, and recurrence monitoring of LUAD.

In this study, the expression and clinical significance of *LDHC*/LDH-C4 in LUAD were discussed using bioinformatics analysis combined with the analysis of the current database, which was further verified by qRT-PCR, IHC, and immunoblotting. The expression and clinical significance of *LDHC* in serum, serum-derived exosome, and tissues from LUAD patients were mutually confirmed with the bioinformatic analysis. The online databases of UALCAN were comprehensively analyzed, which showed that the mRNA and protein levels of LDH-C4 in LUAD were higher than those in the normal control; analysis of the prognostic feature using LOGpc also showed that the differential expression of *LDHC* was negatively correlated with the prognosis of LUAD patients, suggesting that *LDHC* may be a promising biomarker for survival prediction in LUAD.

*LDHC*/LDH-C4 is implicated in the occurrence and development of tumor (10– 12, 15), but there are few studies on *LDHC*/LDH-C4 and lung cancer, especially the detection of serum and exosomal *LDHC* in lung cancer. Exosomes are a kind of single lipid membrane vesicles secreted by cells, with diameters ranging from 30 to 150 nm, which is released into the extracellular matrix after the fusion of intracellular multi-vesicles and cell membrane (23). Exosomes, as a diverse library of molecular substances, contain tumor-related metabolites such as protein, DNA, and RNA to prevent their degradation in circulation (24). Therefore, circulating exosomes can be used as liquid biopsy and non-invasive biomarkers. It was found that the content of free nucleic acids in the blood of tumor patients was significantly higher than that of healthy people, and exosome was involved in the transport of these free nucleic acids (25). The obtained data indicated that exosomes can carry *LDHC* molecules and secrete them to the peripheral blood of BC and HCC patients (13, 14). This study confirmed that *LDHC* exists in the serum of LUAD patients, the positive rate was as high as 66%, and the diagnostic AUCs were higher than 0.80. This study also found the expression of *LDHC* in serum-derived exosome of LUAD patients for the first time, and the two existing forms of *LDHC* (serum and exosomes) have high application value in the diagnosis, efficacy evaluation, and recurrence monitoring of LUAD, which is consistent with *LDHC* expression in BC and HCC verified by previous studies (13, 14). Other previous evidence suggests that the positive rate of expression of seven CTAs in NSCLC tissues was 22%, and the positive rate of *LDHC* in peripheral blood is higher than that of seven CTAs (11), which may be related to the expression form of LDH-C4 and the difference of matrix (serum versus tissue). According to the definition of CTA and the specificity of LDHC/LDHC-C4 expression in somatic cells, *LDHC* should not be expressed in the peripheral blood of normal subjects, but we actually obtained positive results in a small number of healthy subjects, which is an interesting finding. We hypothesize that these healthy individuals with positive serum *LDHC* expression may be at high risk of developing tumors and may be in the early stages of tumors or carcinoma *in situ* that has not yet been clinically diagnosed. Therefore, circulating LDHC has the potential to provide some new clues for early diagnosis of tumors, but further data are needed to confirm this at a later stage.

**Figure 6:**
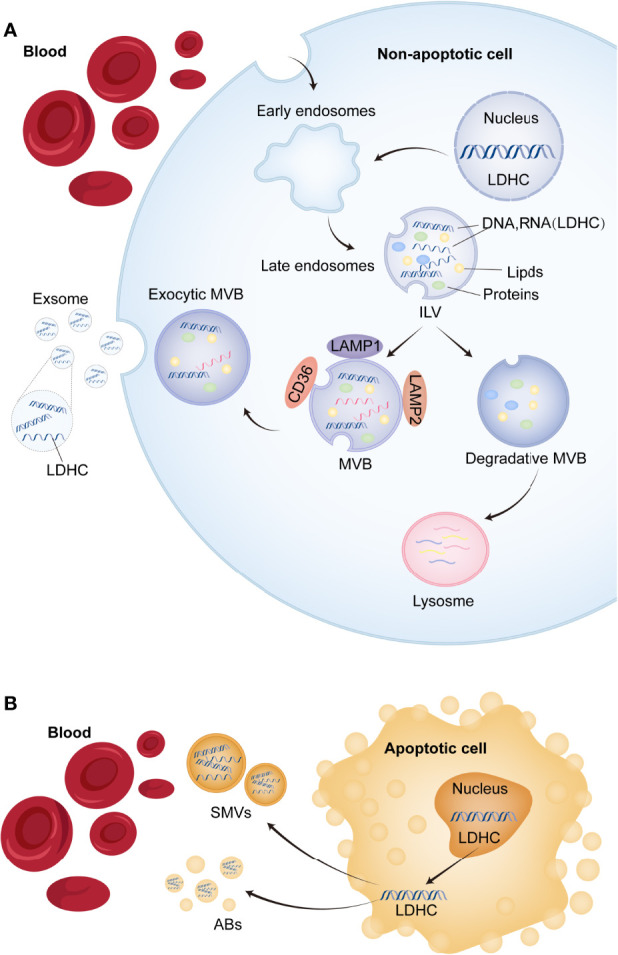
The potential mechanism of exosomal *LDHC* secretion into the circulation. **(A)** The process of releasing exosomes and shed microvesicles (SMVs) (including *LDHC* molecule) into the circulation. Early endosomal contents (proteins and nucleic acids) either circulate into the plasma membrane (PM) or are isolated in intraluminal vesicles (ILVs), which are produced by limiting membrane germination into the lumen of the larger multivesicular body (MVB). **(B)** Apoptotic or non-apoptotic death of tumor cells will also result in the production of apoptotic bodies (ABs). Microbubbles fall off from foamed PM or are released from apoptotic tumor cells. These vesicles are remnants of degraded apoptotic cells and contain nuclear and cytoplasmic components (*LDHC* molecule included).

Currently, studies on the underlying causal links pertaining to the transfer of exosome RNAs into the circulation, as presented in **Figure 6**, conclude that exosome *LDHC* mRNAs might stem from non-apoptosis and apoptosis cells: exosomal *LDHC* molecules might be released into the exocellular median through the exocytic fusion of multi-vesicular bodies (MVBs) with plasmatic membranes (26), before the budding of vesicles straight from plasmatic membranes and micro-vesicles (involving *LDHC*) shedding (**Figure 6A**); the shed micro-vesicles (SMVs) from apoptosis-related cells are referred to as apoptotic blebs (ABs) that contain *LDHC* molecules (**Figure 6B**). Herein, the high expression of *LDHC* in LUAD tissular samples reveals its cancer promoter roles in LUAD, which is substantiated by the poorer OS of LDH-C4-positive sufferers. The expression of *LDHC* mRNAs is also identifiable in serum and serum-originated exosomes of NPC cases. Hence, our team speculated that the cancer promoter *LDHC* molecules were released from exosomes to the outside of cells to facilitate the proliferative ability and growth of oncocytes, and that *LDHC* might exert an effect on the micro-environment of cancer metabolic activity. Kanlikilicer et al. discovered that the expression of endocellular *miRNA-6126* was regulated downward in ovarian cancer cells through exosome transportation, hence activating the expression of the targeted gene *ITGB1* (integrin sub-unit β1) as fuel to the proliferative and invasive abilities of oncocytes (27). Evidently, the assumption in the present research coincides with those discoveries. Nevertheless, as the accurate causal links regarding the exosome secretion of nucleic acid into the circulation are still elusive, further studies in this regard remain warranted.

At the protein level, based on high-throughput LUAD tissue microarray combined with IHC, this study detected the expression level of LDH-C4 protein in LUAD tissues and corresponding adjacent normal tissues of 92 patients with LUAD. It was clear that LDH-C4 protein was mainly expressed in the cytoplasm of LUAD cells, which was consistent with its expression and localization in BC and HCC (13, 14). The positive expression rate of LDH-C4 protein was 96.7% (89/92) in LUAD tissues and 22.6% (19/84) in adjacent normal tissues. However, we found that the expression of LDH-C4 in LUAD tissues had no correlation with patient age, gender, tumor size, lymph node metastasis, clinical stage, and *EGFR* gene mutation. This result is not consistent with *LDHC* expression in serum. The reasons are as follows: Firstly, LDH-C4 was scored according to its IHC score, and circulating *LDHC* expression was quantified using the 2^−ΔΔCt^ method. Secondly, there are patients with high expression and with low expression, of which only 16 cases are included in patients with low expression, and there is a certain bias in the data due to sample size, which may lead to no difference in the analysis results. Thirdly, we only detected fragments of the *LDHC* gene; thus, mRNA may not reflect the C subunit expression, as the linear connection between mRNA and protein expression levels is believed to be only approximately 40% to 50% (28).

The LDH-C4 expression in tumor tissues is related to the prognosis of patients, serving as one of the potential prognostic indicators of malignant tumors (12–14). Our previous study found that the LDH-C4 level in plasma and exocrine is elevated in patients with BC, which can be used to evaluate drug efficacy and to monitor tumor recurrence, and is closely related to OS in BC patients (14). The role of LDH-C4 in renal cell carcinoma has been established with evidence indicating that the survival rate of patients with a positive expression of LDH-C4 is significantly lower than that of patients with negative expression, suggesting the poor clinical prognosis (12). This study found that the 5-year survival rates of patients with a high expression and a poor expression of LDH-C4 were 22.37% and 56.25%, respectively, which indicated that LDH-C4 expression was an independent factor affecting OS in LUAD patients. The results were consistent with previous studies (12–14).

There are many deficiencies in this study: First of all, the sample size was small, especially the number of LUAD patients with a low or negative expression of LDH-C4, including *EGFR* and *ALK* mutation. Therefore, it was impossible to analyze the correlation between LDH-C4 expression and *EGFR* mutation. Secondly, the case information provided by the chip was relatively limited, such as the lack of molecular detection result, including programmed death receptor or ligand (PD-1/PD-L1), and related treatment information. Finally, the type of lung cancer detected was single, and whether LDH-C4 expression in lung squamous cell carcinoma is consistent with LUAD should also be discussed in depth.

In conclusion, this study investigated the expression of *LDHC*/LDH-C4 in tissues, serum, and serum-derived exosomes of LUAD patients. Based on observations and evaluations made during the study, it was suggested that *LDHC*/LDH-C4 served as a better biomarker for clinical diagnosis, efficacy monitoring, recurrence, and prognosis evaluation of LUAD. However, there are inevitable deviations in the conclusions of this study, which need to be further confirmed by large-sample data in the future.

## Data Availability Statement

The datasets presented in this study can be found in online repositories. The names of the repository/repositories and accession number(s) can be found in the article/**Supplementary Material**.

## Ethics Statement

The studies involving human participants were reviewed and approved by the Ethics Committee of Fujian Cancer Hospital. Written informed consent for participation was not required for this study in accordance with the national legislation and the institutional requirements.

## Author Contributions

ZC and YC conceived and designed the study. WP and JC performed the experiments. GS and YX helped to analyze the data. WP wrote the manuscript, and ZC proofed the manuscript. All authors contributed to the article and approved the submitted version.

## Funding

This study was sponsored by the Fujian Provincial Health Technology Project (Grant number: 2019-1-9) and Science and Technology Program of Fujian Province, China (Grant number: 2018Y2003).

## Conflict of Interest

The authors declare that the research was conducted in the absence of any commercial or financial relationships that could be construed as a potential conflict of interest.

## Publisher’s Note

All claims expressed in this article are solely those of the authors and do not necessarily represent those of their affiliated organizations, or those of the publisher, the editors and the reviewers. Any product that may be evaluated in this article, or claim that may be made by its manufacturer, is not guaranteed or endorsed by the publisher.

## Abbreviations

ABs, apoptotic blebs; ALK, anaplastic lymphoma kinase; AJCC, American Joint Committee on Cancer; AUC, area under the curve; BC, breast cancer; CTA, cancer-testis antigen; Cyto C, cytochrome C; EGFR, epidermal growth factor receptor; HCC, hepatocellular carcinoma; HR, hazard ratio; IHC, immunohistochemistry; LDH-C4, lactate dehydrogenase C4; LUAD, lung adenocarcinoma; NSCLC, non-small cell lung cancer; OS, overall survival; PFS, progression-free survival; qRT-PCR, quantitative real-time PCR; SMVs, shed micro-vesicles; TEM, transmission electron microscope; WB, Western blotting.
