# Supplementary material for: Cancer-Testis Antigen LDH-C4 in Tissue, Serum, and Serum-Derived Exosomes Serves as a Promising Biomarker in Lung Adenocarcinoma
Source: Front Oncol. 2022 Jun 24;12:912624. doi: 10.3389/fonc.2022.912624 (PMC9263124; doi:10.3389/fonc.2022.912624)
Supplement: Supplementary file 1 [file Image_1.pdf]

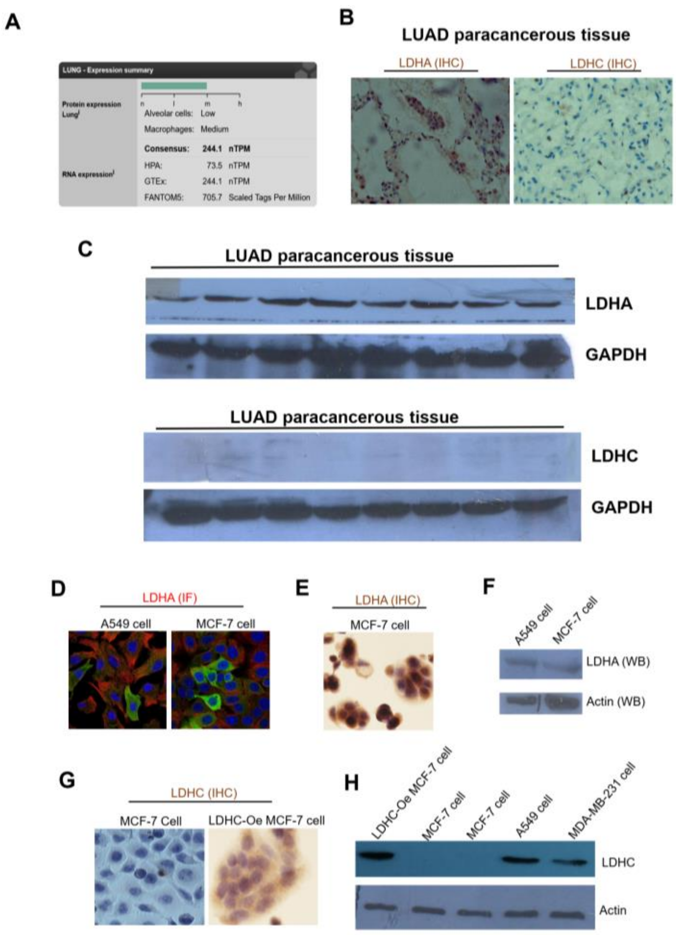


FigureS1. Comparison of specificity between LDHA and LDHC antibodies

The Human Protein Atlas (HPA) database showed that LDHA was expressed at moderate level in normal lung tissue (FigS1A); Rabbit monoclonal [EP1746Y] to LDHC (Abcam; cat no. ab52747) and mouse monoclonal [98A-1F9BB1] to LDHA (Abcam; cat no. ab135396) from Abcam Company were used for IHC. The expression of LDHA and LDHC in para-carcinoma tissues of 84 patients with LUAD was detected (HLugA180Su05; SHANGHAI OUTDO BIOTECH, China). The results showed that the positive rates of LDHA and LDHC in para-carcinoma tissues of LUAD reached 100% (84/84) and 22.6% (19/84), respectively. Figure S1B showed that LDHA (cat no. ab135396) was positive in the para-carcinoma tissues of LUAD, while LDHC (cat no. ab52747) was mostly negative in the para-carcinoma tissues of LUAD. Immunoblotting with the same antibody was used to detect the expression of LDHA and LDHC in the para-carcinoma tissues of 8 cases with LUAD, and the results showed that the

expression of LDHA in the para-carcinoma tissues of 8 cases was positive, but the expression of LDHC was not almost detected (Figure S1C).

The specificity of LDHA and LDHC antibodies was further compared with cell lines. HPA database showed that LDHA was highly expressed in A549 and McF-7 cells (Figure S1D red fluorescence); MCF-7 breast cancer cells were selected for further verification. LDHA protein was expressed, not LDHC protein (see PMID: 26935238) in MCF-7 cells. The results of IHC and immunoblotting experiments with (cat no. ab52747) antibody confirmed that LDHC expression in MCF-7 was negative, while LDHA (cat no. ab135396) antibody was positive (Figure S1 D-H). By contrast, the expression of LDHC in MCF-7 cells could be detected by IHC (Figure S1G)
